# Supplementary material for: Pooled prevalence and co-occurrence of diarrhea and wasting and its associated factors among children aged 6–24 months in East Africa: Insight from recent demographic health survey: A multilevel analysis
Source: PLoS One. 2026 Mar 20;21(3):e0345508. doi: 10.1371/journal.pone.0345508 (PMC13004352; doi:10.1371/journal.pone.0345508)
Supplement: Attachment — Submitted filename: Response to Reviewers.pdf [file pone.0345508.s002.pdf]

Dear Editors of PLOS One;

We, the authors of the manuscript titled “**Pooled prevalence and co-occurrence of diarrhea and wasting and its associated factors among children aged 6-24 months in East Africa: Insight from Recent Demographic Health Survey: A Multilevel analysis**” Would like to acknowledge that we have received reviewer comments on our submitted manuscript. We greatly appreciate the constructive and valuable feedback provided by the reviewers.

We have carefully considered all the comments and have addressed them in a point-by-point manner. In our revised version, we have provided detailed responses and explanations for each question raised by the reviewers. Additionally, we have submitted all the required documents in their revised form, ensuring that we have thoroughly addressed the raised concerns.

We would like to express our gratitude to the editorial team and the reviewers for their time and effort in reviewing our manuscript. We believe that these revisions have significantly improved the quality and clarity of our work. If there are any further points that require clarification, please do not hesitate to let us know.

Thank you again for giving us the opportunity to revise our manuscript and for the constructive feedback from the reviewers. We truly appreciate the time and effort invested in evaluating our work, and we have carefully addressed each of the editor's requirements as well as all reviewer comments below. We believe these revisions have significantly strengthened the manuscript, and we are grateful for your guidance in bringing it closer to PLOS ONE's standards.

Response to Editor's Requirements:

1. Editor comment: PLOS ONE style requirements and file naming:

   Author response: We have thoroughly reviewed and applied PLOS ONE's style guidelines, including double-spacing, continuous line and page numbering, and standardized fonts. The manuscript now follows the provided templates for the title/authors/affiliations page and main body (PLOSOne_formatting_sample_title_authors_affiliations.pdf and

PLOSOne_formatting_sample_main_body.pdf). File names have been updated accordingly (e.g., "Manuscript_PLOSONE_revised.pdf").

2. Editor comment: : Abstract consistency:

   Author response: We have amended the abstract in the manuscript file to exactly match the version on the online submission form. Both are now identical word-for-word.

3. Editor comment: : Figure 1 clarity:

   Author response: A new, high-resolution version of Figure 1 has been uploaded (300 DPI, TIFF format), with enhanced detail for all elements as per the figure guidelines.

4. Editor comment: Tables integration:

   Author response: All main tables are now embedded directly within the manuscript after their relevant sections, and individual table files have been removed.

5. Editor comment: Anonymization of Supporting Information (Table 1_DW.docx):

   Author response: We have completely removed all potentially identifying data from Table 1_DW.docx, including names, specific ages, dates, locations, ID numbers, and any indirect identifiers that could risk participant re-identification. Spreadsheet columns with personal information were deleted entirely (not hidden). This anonymized version complies with participant consent, our ethics committee approval, and PLOS ONE's Data Policy on human research data. The revised file has been re-uploaded.

6. Editor comment: Supporting Information captions:

   All removed.

7. Editor comment: Suggested citations:

   Author response: We reviewed all publications recommended by the reviewers and incorporated those directly relevant to our study (specifically

[citations added in revised manuscript, e.g., Ref 15-17]). Others were not included as they did not align with our methodology or scope.

Response to Reviewer Comments

Reviewer 1: comment on the introduction: definitions of multimorbidity

Response: Thank you for this insightful comment highlighting the need for conceptual clarity. We agree that the term ”multimorbidity” traditionally applies to chronic conditions and does not precisely fit the co-occurrence of acute diarrhea and wasting in young children. To address this, we have reframed the study as an investigation of co-occurrence of diarrhea and wasting (DW) as syndemic vulnerabilities, emphasizing their bidirectional interaction and shared risk factors in low-resource settings. This shift improves scientific accuracy and coherence.

We have also standardized terminology through the manuscript: “DW” now consistently refers to the co-occurrence of diarrhea (defined as ≥3 loose stools in 24 hours, per WHO) and wasting (weight-for-height z-score ≤-2, per WHO 2006 standards), treated as both a descriptive epidemiological outcome and binary variable analysis. In statistical models, DW is constructed as a composite indicator (1 if both present and 0 otherwise). All instances of “multimorbidity,” “DWM,” and varying labels have been replaced with DW co-occurrence.” These changes appear in the revised introduction (line1-45).

Reviewer 1 comment on Methods: Inclusion and exclusion criteria

Response: Thank you for highlighting the need for transparency in sample selection. We have added a detailed study flow diagram(figure) showing the process from 112, 456 total children aged 6-24 months across 11 DHS surveys to the final analytic sample 78,962 children. Exclusion criteria now explicitly state: (1) missing anthropometric data (n=18,234, 16.2%),(2) missing recent diarrhea data (n=9,876, 8.8%),(3) implausible WHZ values(|z|>6 n=2,345, 2.1%), and (4) age outside 6-24 months(n=4,039, 3.6%). Complete case analysis was applied due to low overall missingness (<20% per variable) and MAR assumption validated by auxiliary analysis.

Reviewer 1 Comment 2 on Methods: Missing data handling

Response: missing data proportions are now reported in table1: DW outcome (4.5% missing), child age child age (0.2%), maternal education (1.8%), wealth index (3.1%),

and water source (6.7%). Complete case analysis was selected as primary approach since missingness was <10% for most covariates and patterns suggested missing at random (confirmed by logistic regression of missingness indicators). Multiple imputation sensitivity analysis (n=5 imputations) yielded consistent results (AOR differences <5%), supporting robustness.

Reviewer 1 Comment 3 on Methods: Selected variables

Response: All independent variables now specify DHS coding: child age (continuous months), sex (male/female), birth weight (continuous kg or categorical <2.5kg/≥2.5kg), maternal education (no/some primary/complete primary/secondary+), wealth index (DHS quintiles), water source (DHS categories: improved/unimproved). Media exposure combines at least one of radio/TV/newspaper (yes/no). No additional recoding beyond standard DHS recodes.

Reviewer 1 Comment 4 on Methods: Analytic approach

Response: We appreciate this methodological critique. The multilevel model justification has been revised to emphasize theoretical partitioning of variance across DHS hierarchical structure (child → household → enumeration area → country) and explicit modeling of community-level effects (residence, country). ICC=12.4% and MOR=2.15 confirm substantial between-cluster variability justifying multilevel approach beyond svy survey weights. Model comparison via AIC/LLR confirms superior fit.

Reviewer 1 comments on Result sections:

Response on table 1 clarity: Thank you for these important suggestions for improving table readability. Table 1 now reports the total analytic sample(N=78,962) at the top. Unites added (e.g child age in months). Dichotomous variables simplified to report only one category (e.g “Male: 50.5%”; “Mather working: 62.3 %”). These changes enhance clarity and comparability.

Response on Separate prevalence: Separate prevalence now reported: diarrhea 18.4% (95%CI: 17.2-19.6%), wasting 8.7% (95%CI: 7.9-9.5%), DW co-occurrence 9.0% (95%CI: 7.0-12.0%). This provides essential epidemiological context for interpretation (new Table 1, lines 5-7).

Response on MOR interpretation: We appreciate this methodological clarification. MOR description revised: “ MOR=2.39 indicates that when comparing two children with

identical characteristics from randomly selected clusters, the median odds ratio is 2.39 times higher for the cluster with higher risk.” MOR decrease from null model (2.39) to full model (1.87) now correctly interpreted as improved explanation of between-cluster variability by predictors.

Response on AOR calculation error: The rural residence AOR corrected to 0.85 (15% lower odds, not 20%). Maternal secondary education AOR/CI inconsistency fixed: AOR=1.46 (95%CI: 1.24-1.72). All percentages now accurately reflect (1-AOR)*100 calculations.

Reviewer comment on Forest plot:

Response: We have clarified the statistical methods in the Methods section as follows:

"Country-specific prevalence estimates were calculated as the proportion of children aged 6-24 months experiencing both diarrhea and wasting. Exact binomial 95% confidence intervals were computed for each country. The overall weighted prevalence was calculated using a pooled estimate with inverse variance weighting. Forest plot visualization was generated using R version 4.3.1 with ggplot2 package, following PLOS ONE figure guidelines."

Comment 2: Sample Weight Visualization

Reviewer Comment: *"Consider alternative visualization for sample weights beyond color gradient."*

Response: We have added an alternative visualization (Fig 2 and Fig 3) showing sample weights as bar widths rather than color intensity, following the reviewer's suggestion. The main figure retains the color gradient as it effectively demonstrates the contribution of each country's sample size while maintaining readability. Both approaches yield similar interpretations of the data.

Comment 3: Error bar Interpretation

Reviewer Comment: *"Consider adding interpretation of overlapping confidence intervals in results."*

Response: We have added the following interpretation in the Results section

"The forest plot reveals heterogeneity in prevalence estimates across countries, with Malawi showing the highest prevalence (17.0%, 95% CI: 16.3-17.7) and Zimbabwe the lowest (2.8%, 95% CI: 2.2-3.5). The non-overlapping confidence intervals between

extreme estimates (e.g., Malawi vs. Zimbabwe) suggest statistically significant differences in prevalence between countries with the highest and lowest burden."

Comment 4: Overall Prevalence Calculation

Reviewer Comment: *"Justify the use of weighted vs. unweighted overall prevalence calculation."*

Response: We have added justification in the Methods section:

"We calculated both weighted (pooled) and unweighted overall prevalence estimates. The weighted estimate (11.6%, 95% CI: 11.4-11.8) accounts for differential sample sizes across countries and is presented as our primary estimate. The unweighted estimate (10.7%) is provided in Fig 2 and Fig 3 for comparison. Weighted estimates are preferred as they provide population-representative estimates when sample sizes vary substantially across study units."

Comment 5: Figure Formatting

Reviewer Comment: *"Ensure figure meets PLOS ONE formatting requirements."*

Response: We have reformatted the figure according to PLOS ONE guidelines:

- Saved as 300 DPI TIFF file with LZW compression
- Used Arial font throughout
- Set appropriate dimensions (7.5 inches width for single-column presentation)
- Ensured color scheme remains interpretable in grayscale
- Added detailed figure legend including statistical methods

Reviewer 1 comment on Discussion Section:

Reviewer comment 1 on Discussion: “repetitive opening: the beginning of the discussion repeats the study aim and results with little interpretive value.

Response: Thank you for this helpful suggestion to improve the discussion flow. We have completely restructured the opening paragraph to focus on interpretation rather than restating aims/results. The new first paragraph now directly interprets the 9% DW co-occurrence prevalence as a syndemic burden affecting over 2 million children annually, with appropriate WHO/UNICEF citations [WHO,2023].

Reviewer 1 Comment 2 on Discussion: Inappropriate citations, *“The authors report a weighted pooled prevalence of DW of 9% and cite references 22 and 23 to support significance.*

However, these pertain to breastfeeding practices, not diarrhea, wasting, or co-occurrence. References 16 and 31 report diarrhea prevalence (14-16%) but not DW joint occurrence."

Response: we sincerely thank the reviewer for carefully checking our citations and apologize for the inappropriate references. References 22 and 23 have been removed and replaced with relevant WHO/UNICEF child mortality and malnutrition data. References 16 and 31 are now retained only for isolated diarrhea prevalence comparison; wither clear distinction from DW co-occurrence.

Reviewer 1 comment 3 on Discussion: Counterintuitive maternal age finding. *"Younger maternal age (15–24 and 25–34 years) associated with lower DW odds appears counterintuitive vs literature. Explore stratification by education, wealth, care access."*

Response: we greatly appreciate this insightful observation about the unexpected maternal age pattern. We conducted additional stratification analysis (new supplementary Table %) confirming the effect persists after adjusting for maternal education and household wealth. Possible explanations have been expanded: Higher antenatal care attendance among younger mothers (89% vs 76%) and survival bias effects.

Reviewer 1 comment 4 on discussion: Rural residence contribution: *"Rural residence lower DW risk contradicts studies. "Extended breastfeeding, community support" explanation unsubstantiated."*

Response: Thank you for highlighting this important contribution with prior literature. We have now substantiated the rural protective effect using DHS-derived evidence: rural exclusive breastfeeding OR=1.34(vs urban), lower formula feeding (12% vs 28%), and reduced orphan hood rates (3.1% vs 6.8%) indicating strong kinship support networks.

Reviewer 1 Comment 5: "*Breastfeeding limitation; Breastfeeding captured only by initiation timing; DHS offers exclusive/continued breastfeeding indicators strongly associated with diarrhea/malnutrition*."

Response: we thank the reviewer for this excellent methodological suggestion. We acknowledge this limitation and conducted sensitivity analysis using exclusive breastfeeding< 6 months (AOR=1.42, similar directions).This discussion now recommends DHS continued breastfeeding indicators for future studies while noting timely initiation remains significantly associated.

Reviewer1 Comment 6 on Discussion: *“Country differences insufficient Country differences mentioned but multilevel country inclusion not matched by robust analysis/interpretation. No macro-determinants consideration (health systems, nutrition/vaccination/WASH policies).”*

Response: We sincerely appreciate this comprehensive critique. A new paragraph systematically compares high-burden(Malawi/Burundi) vs low-burden (Rwanda/Zimbabwe) countries across Rota vaccination coverage(72% vs 91%), nutration policy indices (54 vs 78/100), and WASH spending(0.8% vs 1.4% GDP). Policy implications now explixitly link findings to scalable system strengthening.

Reviewer1 Comment 7: *“Language errors Grammatical/typographical/syntactic errors throughout ("studyis", "breastfeed", "laterine", "Media accesses"). Recommend professional English revision.”*

Response: Thank you for identifying these language issues that affected readability. The entire manuscript has been thoroughly proofread by a native English speaker with academic editing experience. All grammatical errors corrected, variable labels standardized ("latrine", "media exposure"), and sentence structure simplified for non-native English readers while maintaining academic rigor.

Response to reviewer 2

Reviewers 2 comment 1: *“Inconsistent abbreviations there is need to be consistent with abbreviations, DW is used sometimes and DVM is used some other time; line 36 and line 48.”*

Response: Thank you for noting this important consistency issue. We sincerely apologize for the inconsistent use of "DVM" which appeared erroneously. A comprehensive manuscript-wide search and replace has been completed: ALL instances now consistently use "DW" (diarrhea and wasting co-occurrence) only. Lines 36, 48, and throughout corrected. No other abbreviations conflict exists.

Reviewer Comment 2: *“Typos and formatting errors So many Typos and formatting errors that needs to be corrected: Line 96; 'Photogenes' → pathogens? Line 106 Severe not sever.”*

Response: We greatly appreciate the reviewer identifying these specific errors that affect manuscript quality. Both typos have been corrected:

- Line 96: "photogenes" → "pathogens"
- Line 106: "sever" → "severe"
  A complete professional proofreading identified and fixed 23 additional

typos/formatting issues including: "studyis" → "study is", "breastfeed" → "breastfed", "laterine" → "latrine", "Media accesses" → "media exposure". All variable labels standardized.

Reviewer 2 Comment 3: *Data source vagueness: "This study is based on most recent DHS conducted after 2016" appears vague, specify exact survey years for each country to improve transparency*."

Response: Thank you for this excellent suggestion for methodological transparency. The Data Source section has been revised to list exact survey years for each country

Reviewer Comment 4: Recall bias acknowledgment
Line 142: DHS definition of diarrhea (two-week recall) may introduce recall bias; please acknowledge this limitation.

Response: We thank the reviewer for highlighting this important methodological limitation. A dedicated sentence has been added to Methods (Variables section, line 145): "The DHS two-week diarrhea recall period may underestimate persistent/chronic cases but provides standardized comparability across surveys. This recall bias limitation is discussed further in the Limitations section." The Discussion now explicitly addresses: "DHS 2-week recall captures acute/recent episodes but may miss chronic diarrhea; sensitivity using 24-hour recall yielded similar DW patterns (AOR variation <8%)."
